# Multifaceted case management during pregnancy is associated with better child outcomes and less fetal alcohol syndrome

**DOI:** 10.1080/07853890.2023.2185808

**Published:** 2023-03-15

**Authors:** Philip A. May, Anna-Susan Marais, Wendy O. Kalberg, Marlene M. de Vries, David Buckley, Julie M. Hasken, Cudore L. Snell, Ronel Barnard Röhrs, Dixie M. Hedrick, Heidre Bezuidenhout, Lise Anthonissen, Erine Bröcker, Luther K. Robinson, Melanie A. Manning, H. Eugene Hoyme, Soraya Seedat, Charles D. H. Parry

**Affiliations:** a Nutrition Research Institute, The University of North Carolina at Chapel Hill, Kannapolis, NC, USA; b Department of Psychiatry, Faculty of Medicine and Health Sciences, Stellenbosch University, Cape Town, South Africa; c Center on Alcohol, Substance Abuse and Addictions, The University of New Mexico, Albuquerque, NM, USA; d School of Social Work, Howard University, Washington, DC, USA; e Department of Pediatrics, State University of New York, Buffalo, NY, USA; f Department of Pathology and Pediatrics, Stanford University School of Medicine, Stanford, CA, USA; g Sanford Children’s Genomic Medicine Consortium, Sanford Health, Sioux Falls, SD, USA; h Alcohol, Tobacco and Other Drug Research Unit, South African Medical Research Council, Cape Town, South Africa

**Keywords:** Fetal alcohol spectrum disorders (FASD), fetal alcohol syndrome (FAS), prenatal alcohol exposure (PAE), multifaceted case management (MCM), prevention, intervention, prenatal alcohol use, maternal risk for FASD

## Abstract

**Background:**

Pregnant women participated in multifaceted case management (MCM) to prevent Fetal Alcohol Spectrum Disorders (FASD).

**Methods:**

Women recruited from antenatal clinics for a longitudinal child development study were screened for alcohol use. Forty-four pregnant women were defined as high-risk drinkers on the Alcohol Use Disorder Identification Test (AUDIT) by an AUDIT score ≥8 and participated in 18 months of MCM to facilitate reduction or cessation of alcohol consumption. Forty-one women completed MCM. Fifty-five equally high-risk women who received standard antenatal care comprised the comparison/control group. Development in offspring was evaluated by a blinded interdisciplinary team of examiners through 5 years of age.

**Results:**

At five years of age, more children (34%) of MCM participating women did not meet the criteria for FASD *vs.* non-MCM offspring (22%). Furthermore, a statistically significant (*p* = .01) lower proportion of MCM offspring (24%) was diagnosed with fetal alcohol syndrome (FAS) compared to controls (49%). Children of MCM participants had significantly (*p* < .05) better physical outcomes: lower total dysmorphology scores, larger head circumferences, longer palpebral fissures, and higher midfacial measurements. Neurodevelopment results showed mixed outcomes. While Bayley developmental scores indicated that MCM offspring were performing significantly worse on most domains through 18 months, group scores equalized and were not significantly different on Kaufman Assessment Battery neurobehavioral measures by five years. Regression analyses indicated that offspring of women who received standard antenatal care were associated with significantly more negative outcomes than MCM offspring: a diagnosis of FAS (OR = 3.2; 95% CI: 1.093–9.081), microcephaly (OR = 5.3; 95% CI: 2.1–13.5), head circumference ≤10th centile (OR = 4.3; 95%CI: 1.8–10.4), and short palpebral fissures (OR = 2.5; 95% CI: 1.0–5.8).

**Conclusion:**

At age five, proportionally fewer children of MCM participants qualified for a diagnosis of FAS, and proportionally more had physical outcomes indicating better prenatal brain development. Neurobehavioral indicators were not significantly different from controls by age five.

**KEY MESSAGES**
- Multifaceted Case Management (MCM) was designed and employed for 18 months during the prenatal and immediate postpartum period to successfully meet multiple needs of women who had proven to be very high risk for birthing children with fetal alcohol spectrum disorders (FASD).
- Offspring of the women who participated in MCM were followed up through age five years and were found to have significantly better physical outcomes on multiple variables associated with fetal alcohol syndrome (FAS) and FASD, such as larger head circumferences and fewer minor anomalies, than those children born to equally at-risk women not receiving MCM.
- Fewer children of women receiving MCM were diagnosed with FASD than the offspring of equally-at-risk controls, and significantly (*p* = .01) fewer MCM offspring had FAS, the most severe FASD diagnosis.

## Introduction

The prevalence of fetal alcohol syndrome (FAS) and fetal alcohol spectrum disorders (FASD) in some communities in the Western Cape Province (WCP) of South Africa (ZA) is the highest documented in the published literature anywhere to date [1]. FASD is the umbrella term for a continuum of adverse effects due to prenatal alcohol exposure. Individuals who fall within the FASD continuum present with physical, neurocognitive, and behavioral impairments including, but not limited to, growth deficiencies, minor physical anomalies, and deficiencies in at least one cognitive or behavioral domain. The adverse effects of prenatal alcohol exposure are persistent across the lifespan [2,3]. In the WCP, FASD affected 17–31% of first grade students in the general populations of five communities [4–7]. The prevalence of FASD in general populations has been conservatively estimated to be 1–5% in the United States, 2–3% in Ontario, Canada, 2–4% in Italy, 1.8% in Manchester, United Kingdom, and 4–6% in Croatia [8–14]. FASD prevalence has also been reported as high elsewhere in ZA and to vary by ethnicity, socioeconomic status, and community of residence [15–17].

### Preventing FASD

Prenatal alcohol exposure (PAE) presents the most serious overall risk to the fetus of any commonly used recreational substance. The National Institute of Alcohol Abuse and Alcoholism (NIAAA) defines binge drinking as a pattern of drinking that results in a blood alcohol concentration of 0.08% or greater; in females this equates to 4 or more drinks in about 2 h [18]. However, previous research on risk to fetus has indicated that binge drinking three or more standard drinks per occasion poses a significant risk factor for FASD or other negative physical or neurobehavioral outcomes in many populations [1,19–23]. Thirty-seven to 60% of women in European countries, and over 10% of women in the United States (US) reported alcohol use during pregnancy [24]. Binge drinking in any and all trimesters is particularly harmful [25]. One-third of Australian women reported prenatal drinking, and 18.5% from varying social strata reported first trimester binge drinking, particularly on special occasions [26]. Globally, estimates indicate that 9.8% of women drink, and up to 14% binge drink prenatally [1,27]. Prenatal drinking was reported as 40–50% in population-based studies of the WCP of ZA, and prenatal binge drinking was more common [28,29] than the 7% in province-wide surveys [30]. In pursuing FASD research in SA, we have been ethically compelled to provide preventive services for as many heavy drinking women as possible with available resources.

### Screening and interventions

For this intervention, Multifaceted Case Management (MCM) employed principles of Motivational Interviewing (MI) and Community-Reinforcement Approach (CRA) in a program that taught basic coping skills for life stresses and techniques for reducing alcohol use [31–36]. The goal was to improve child outcomes, document key developmental outcomes empirically, and prevent FASD by supporting pregnant women through empathic care *via* MCM.

Alcohol screening and brief interventions during pregnancy have been recommended for education and prevention of alcohol-exposed pregnancies [37–39]. Recommended preventive education themes include using positive, carefully-worded questions and conversations to explore drinking habits, building trust with patients, and providing rationale and encouragement for alcohol-free pregnancies [40–43]. Brief, women-focused prenatal interventions that provide basic knowledge of fetal development and FASD, assistance in navigating the healthcare system, education on domestic partner communications/relationships, and enhanced motivation for an alcohol-free pregnancy are also recommended [44–47]. While 81% of US adults report being asked about their drinking by a healthcare provider, only 38% were asked about binge drinking; and of those who binge drink, 42% were advised of harmful drinking levels, and 80% were not advised to reduce drinking [38]. Among pregnant women in Maryland, 19% were not screened for alcohol consumption and 30% indicated that their healthcare provider did not counsel them about the adverse effects of prenatal alcohol exposure [48]. In the National Survey of Family Growth 2011–2013, the CDC found that only 54% of women who reported consuming alcohol 3 months before pregnancy received information on the effects of alcohol on fetal development from their healthcare provider [49]. Overall, there is a pressing need for preventive interventions, such as case management and similar programs for prenatal alcohol use that are not commonly practiced [50].

It is often necessary to adapt general screening and prevention modalities and therapeutic strategies to local cultures. Motivational Interviewing (MI) [33] is a therapeutic change modality implemented to reduce problem drinking for individuals, including pregnant women [51] and specific cultural groups, such as American Indians [52–54]. Furthermore, Rendall-Mkosi et al. [55] reported that a five-session randomized, controlled trial of MI intervention for ZA women of childbearing age significantly reduced alcohol-exposure in pregnancy by 51% compared to 28% in non-participants.

### Underpinnings and practices of multifaceted case management to prevent FASD

Our team has combined the above recommended intervention themes, standard practices, and techniques from MI and the Community Reinforcement Approach (CRA) into MCM for the prevention of FASD within our research programs in ZA. MI uses five core principles to elicit and manage positive changes in alcohol use: (1) express empathy for the client; (2) develop discrepancy in the client’s understanding of current behavior and the need to accomplish important goals; (3) avoid argumentation; (4) roll with client resistance; and (5) support client self-efficacy [33]. CRA provides techniques and guidelines for therapists/managers to recruit, motivate, and guide a client’s family and significant others to elicit positive change in drinking and social behaviors [56].

### Previous evaluations of the MCM FASD prevention

In a previous evaluation of this MCM approach that specifically targeted change in drinking practices, we reported that pregnant women reduced their drinking significantly on: total drinks on weekends 6 months after MCM initiation and had significantly lower estimated peak blood alcohol concentrations after 6 and 18 months [57]. However, reductions in drinking during pregnancy were often followed by postpartum relapses to heavy weekend drinking 18 months later. Scores on the Alcohol Use Disorders Identification Test (AUDIT) indicated that problematic drinking decreased significantly during pregnancy, and remained lower 6–12 months after MCM initiation [58].

### The hypothesis of this evaluation: a focus on child outcomes

While several measures in the previous evaluation of MCM provided evidence of a reduction in prenatal drinking, this study focused on the empirical documentation of specific child outcomes. The hypothesis in this study was whether reduced prenatal drinking resulted in improved indicators of child development and fewer cases of FASD among offspring of women receiving MCM. To our knowledge, measurement of multiple, actual child outcomes (both physical and neurobehavioral) has not been reported before in evaluating an FAS prevention program.

## Methods

### Screening and criteria for inclusion in multifaceted case management

**Figure 1.**
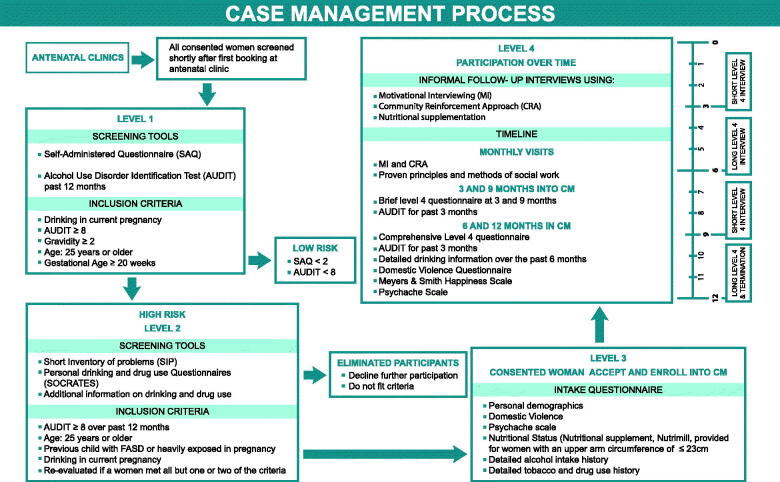
The multifaceted case management (MCM) process for high-risk women in South African fetal alcohol spectrum disorders research.

Project research staff recruited study participants from community health care clinics in two WCP regions separated by a mountain range (Figure 1). All pregnant women registering for antenatal care [mean = 4.7 months gestation (*SD* = 1.8 months)] in both regions were invited to participate in first-level screening for a longitudinal study of child development. The screening included the Self-Administered Questionnaire (SAQ) [59], AUDIT [60], and questions on previously-identified risk factors from previous WCP studies [20,61].

While women were recruited for the development study in both regions, MCM could only be offered in one of the two regions. MCM was provided to women, prenatally and postpartum, for 18 months. The growth and development of offspring were studied 60 months postpartum. Comprehensive, structured maternal risk interviews were conducted with participating MCM women at intake, 3, 6, 9 12, and 18 months after MCM intake (Figure 1).

Criteria for MCM entrance included: (1) self-reported drinking of eight or more drinks per week during the index pregnancy; or (2) one binge of three or more drinks reported any day of the week on the SAQ; or (3) scores of ≥8 on the AUDIT (possible 40 points) at screening.

### Training and implementation of MCM

MCM was implemented by bilingual (Afrikaans and English) project officers formally trained as social workers or nurses. Study protocols and skills utilized in MCM were gained from two separate training workshops, each consisting of two weeks of intensive, specialized instruction from experts in social work, MI, CRA, FASD, and intervention. Professional mentoring and supervision were provided throughout the study by a bi-lingual lead trainer, a native of the WCP with a doctorate in social work and decades of professional experience. The training was implemented to ensure the fidelity of implementation of MCM in the local culture of this predominantly mixed-race, ‘Cape Coloured’ population.

Following recruitment into the study, project officers made visits at least once a month during the remainder of pregnancy with each MCM participant, commencing two weeks after screening. Visits every two weeks were made if circumstances of higher risk warranted. After the children were born, visits were less frequent, and the focus switched to the maintenance of abstinence or alcohol use reductions to facilitate alcohol-free breastfeeding and options for voluntary birth control. Postpartum MCM also addressed themes, such as coping with postpartum depression, family planning, and infant care, delivered empathically.

### Nutritional supplementation for low BMI women

Low maternal weight and body mass index (BMI) were identified previously as important distal risk factors for FASD in these communities [20,62,63]. At intake, the left upper arm circumference was measured to determine the need for nutritional supplements to improve pregnancy outcomes for low BMI participants. Women with an upper arm circumference of ≤23cm (*n* = 22, 50%) were provided supplementation powder for mixing with water for daily consumption during the index pregnancy.

The left upper arm circumference and the women’s weight were measured at each formal interview during the MCM process. These were measured four times in pregnancy and once post-partum.

### MCM investigation was part of the longitudinal study of development

Recruitment into MCM was concurrent with the recruitment of a large cohort (*n* = 199) to test the efficacy of Bayley Scales of Infant Development (BSID-III) [64] for early life detection of children with FASD [65]. All children born from these pregnancies were monitored for growth and development with the same protocols. All participants were informed at the recruitment interview at the first antenatal visit that drinking during pregnancy was harmful to the fetus, and all participants were encouraged to stop drinking or reduce drinking during pregnancy and encouraged to use contraception once the child is born. Throughout the course of the MCM process, participants were provided information on the harmful effects of alcohol on the development of the fetus and given additional information on fetal and child development, the role of the father in pregnancy, contraception, supportive messages, information on resources in the community, and social support to assist abstinence or reduced alcohol use and other MCM support at each visit. Women in the control group received only standard prenatal care and information from clinic personnel.

### Assessing and diagnosing the offspring

In-person examinations and assessments of physical growth and dysmorphology of the children born to the participating women were undertaken at six weeks and 9, 18, 42, and 60 to 72 months by research staff. Final examinations were administered by pediatric dysmorphologists and consisted of measuring height, weight, occipitofrontal (head) circumference (OFC), facial anthropometry, and the assessment of 30 minor anomalies of the face, heart, and hands [66,67].

Developmental assessments with the BSID-III were performed at 6 weeks, 9, 18, and 42 months to assess milestones in cognitive, language, motor, and social-emotional domains. At 60–70 months, the Kaufman Assessment Battery for Children (KABC-II) [68] was administered by psychometrists to provide a mental processing index. The KABC-II is considered a culturally fair test that has been used in previous ZA research [69,70]. Age-adjusted percentile rank scores (range: 1–99) are presented for both BSID-III and KABC. All MCM and non-MCM women completed an in-person, final MCM maternal risk interview at 42 or 60 months postpartum. The interviews covered demographic, socioeconomic, childbearing history, and alcohol, tobacco, and other drug use variables before pregnancy and each trimester. All participants were compensated for their time with credit vouchers.

Final diagnoses for children were assigned in structured, multidisciplinary case conferences in either 2015 or 2017. Results from physical, neurobehavioral, and maternal risk assessments were reviewed by team members who had performed the assessments. All clinicians and interviewers had been blinded to prior information on each child and mother during clinical exams, testing, and interviews. MCM participation status was also blinded throughout the exams, testing, and diagnostic processes, for the overall focus of the parent study was the longitudinal assessment of the larger developmental cohort for child growth and development [65]. Final diagnoses were assigned by the pediatric dysmorphologists using the revised Institute of Medicine (IOM) guidelines [67,71]. FASD diagnoses included: (1) fetal alcohol syndrome (FAS); (2) partial fetal alcohol syndrome (PFAS); (3) alcohol-related neurodevelopmental disorder (ARND); or (4) alcohol-related birth defects (ARBD).

### Sample

**Figure 2.**
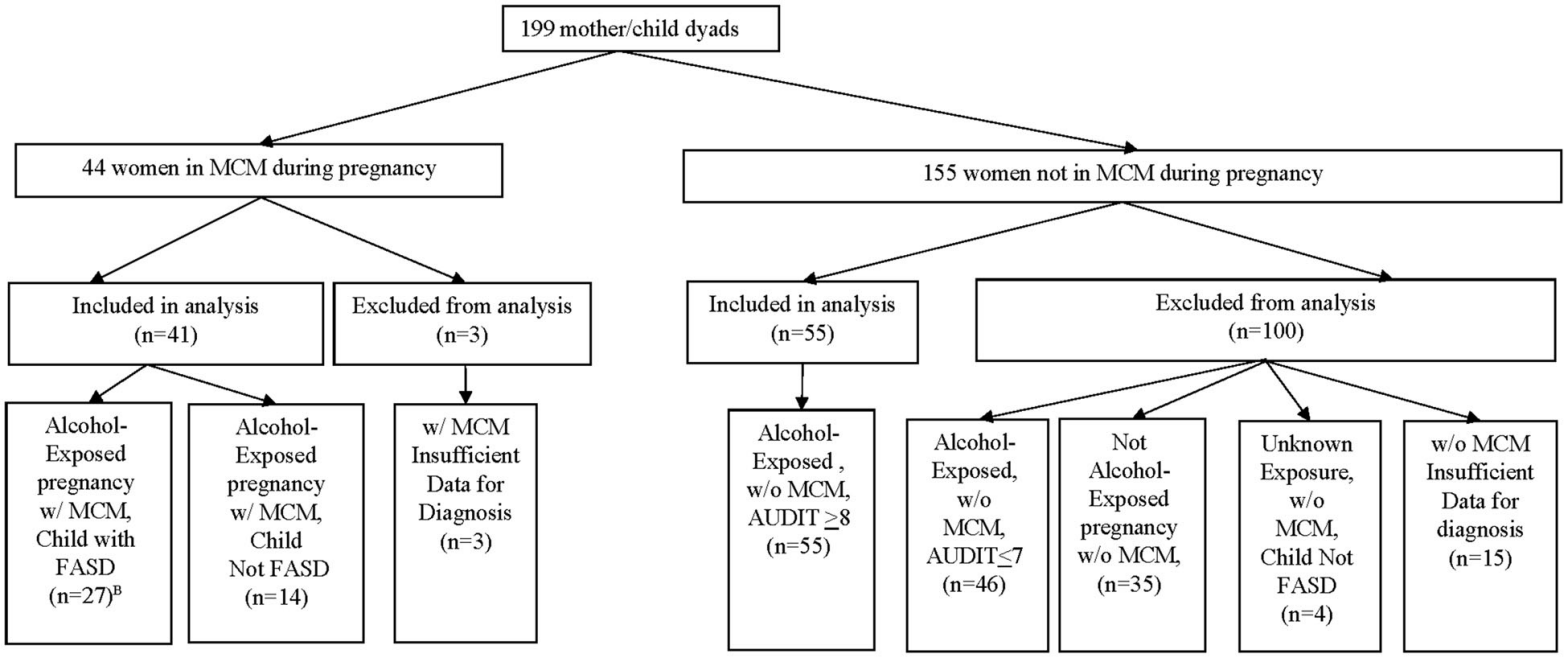
Longitudinal cohort by multifaceted case management participation, pregnancy exposure status, and child diagnosis.^A A^Diagnosis based on either March 2015 diagnosis or October 2017 diagnosis. ^B^Two children met criteria for FAS based on physical dysmorphology and neurocognitive assessments while mother denied alcohol use during pregnancy.

All maternal/child dyads participated in the developmental study from the initial prenatal visit until the children reached a minimum age of 60 months (Figure 2). Forty-four women who met heavy drinking criteria and lived in the region where MCM could be offered were enrolled in MCM, but three women dropped out and were excluded from the analysis. Among the other 155 women and children, 19 dyads had insufficient data to rule on an FASD diagnosis when final diagnoses were made. Of the remaining 136 women, 35 (25.7%) reported abstaining from alcohol, 46 (33.8%) were drinkers with AUDIT scores ≤7, and 55 (40.4%) met the criteria for hazardous drinking. Therefore, the final sample in this MCM evaluation is 96 dyads, 41 of whom were in MCM for 18 months during pregnancy and postpartum, and 55 dyads not participating in MCM who met the criteria for heavy drinking for the index pregnancy.

### Data analysis

Data analysis was completed in SPSS 26.0 [72]. *t*-Tests (for continuous variables) and chi-square tests (for categorical variables) compared maternal demographics and alcohol consumption. The children’s physical and cognitive outcomes at 60 months for the neurobehavioral testing and up to 80 months for age-adjusted physical traits are presented. Given the fact that this evaluation of an FASD intervention *via* a comparison of global child outcomes is the first of its kind, alpha significance levels were set a *p* < .05. Additionally, in interpreting Table 3 results, we also provided Bonferroni-adjusted values. *Post-hoc*, *Z*-tests compared the proportion of children with specific diagnoses within the FASD continuum. Unadjusted binary logistic regression evaluated the association (*p* ≤ .05) of MCM participation on the likelihood of offspring having specific FASD diagnoses (e.g. FAS *vs.* control), small head circumference (≤3rd centile *vs.* not and ≤10th centile *vs.* not), and short palpebral fissures (≤3rd centile *vs.* not) for maternal participants and non-participants and for women who reported drinking in the 3rd trimester. The medical geneticists/dysmorphologists are trained and highly experienced in the differential diagnosis of FASD as one of a range of birth anomalies, and an FASD diagnosis is made only after excluding other birth defects and anomalies common to other phenotypes which are caused by other known genetic or teratogenic influences. Therefore, statistical controls for exposures other than tobacco were not needed. Tobacco has been shown to exert a relatively minor influence on the diagnosis of FASD in this population when compared to the influence of alcohol [4,6], and it is also controlled and assessed separately in the regression analysis.

## Results

### MCM contact with participants

The mean number of MCM contacts with participants was 10.5 (*SD* = ±4.7), and total face-to-face contact time averaged 7.6 h per participant (*SD* = ±2.6 h). Final status assessment visits and data collection with all women (MCM participants and non-participants) occurred 18 months post-recruitment.

### Maternal risk factors of MCM women and controls

Proximal and distal maternal risk factors presented in Table 1 indicate that both groups of women were generally high risk, for AUDIT and T-ACE (Tolerance, Annoyance, Cut Down, and Eye Opener) scores were very high for both. However, women who participated in MCM were historically the highest risk drinkers as measured by the AUDIT (19.7 *vs.* 15.1, *p* = .001) and both groups were equally high risk on the T-ACE (3.5 *vs.* 4.0, *p* = .061). The participants in MCM were entered into the study at the time of the first antenatal visit which was generally in the 2nd trimester (mean = 4.7 months). The usual number of drinks per drinking day (DDD) was significantly higher (*p* ≤ .05) in the non-MCM participant women in the 1st trimester (10.1 *vs.* 6.2) and 2nd trimester (9.9 *vs.* 6.2). Frequency of drinking, however, was significantly higher in the MCM group in the 1st trimester: 92.3% of MCM participants and 71.6% non-participants usually drank 1–7 days every week. In the 2nd and 3rd trimesters, the difference in frequency was not significantly different. There were no significant differences in breastfeeding measures, including the percentage drinking alcohol during the breastfeeding period (66.0 *vs.* 64.7%). Of the distal risk factors, only parity at intake was significant; the parity of non-MCM participants was higher (3.0 and 2.3), indicating a slightly greater risk for FASD. While a higher AUDIT score for the MCM participants indicated historically more problematic drinking and MCM participants reported greater frequency in the 1st trimester, the non-MCM participants had a significantly higher DDD in the 1st and 2nd trimesters. Overall, the known risk factors were equal among the groups; both groups were high-risk.

**Table 1.** Maternal risk factors among alcohol-exposed pregnancies by maternal multifaceted case management participation.

	Alcohol-exposed without MCM (*n* = 55)		Alcohol-exposed with MCM (*n* = 41)			
	*N*	Mean (*SD*)	*N*	Mean (*SD*)	*p*	
Proximal risk factors						
T-ACE at intake	55	3.5 (1.3)	40	4.0 (1.1)	.061	
AUDIT score at intake	55	15.1 (5.5)	41	19.7 (7.5)	.001	
Estimated Month Enrolled in MCM	–	–	41	4.7 (1.8)	–	
Drank in 1st trimester (% Yes)^a^	48/50	96.0	36/38	94.7	.778	
Usual number of drinks per drinking day—1st trimester	49	9.7 (11.0)	38	5.9 (3.9)	.046	
Usual number of drinks per drinking day—1st trimester^b^	49	10.1 (11.1)	38	6.2 (3.7)	.047	
Usual frequency of drinking—1st trimester						
Every day or almost every day	5	9.4	0	0.0		
3–4 times per week	14	26.4	19	48.7		
1–2 times per week	19	35.8	17	43.6		
1–3 times per month	8	15.1	0	0.0		
1–2 times per 3 months	2	3.8	0	0.0		
Unknown frequency	3	5.7	1	2.6		
Did not drink	2	3.8	2	0.0	.020	
Drank in 2nd trimester (% Yes)^a^	37/50	74.0	33/38	86.8	.139	
Usual number of drinks per drinking day—2nd trimester	46	7.1 (9.1)	38	5.4 (4.5)	.284	
Usual number of drinks per drinking day—2nd trimester^b^	46	9.9 (9.3)	38	6.2 (4.2)	.040	
Usual frequency of drinking—2nd trimester						
Every day or almost every day	4	7.5	0	0.0		
3–4 times per week	10	18.9	14	35.9		
1–2 times per week	16	30.2	17	43.6		
1–3 times per month	4	7.5	2	5.1		
1–2 times per 3 months	1	1.9	0	0.0		
Unknown frequency	5	9.4	1	2.6		
Did not drink	13	24.5	5	12.8	.098	
Drank in 3rd trimester (% Yes)^a^	24/49	49.0	20/38	52.6	.735	
Usual number of drinks per drinking day—3rd trimester	49	5.8 (11.7)	37	2.6 (4.1)	.117	
Usual number of drinks per drinking day—3rd trimester^b^	49	11.8 (14.6)	37	5.0 (4.5)	.059	
Usual frequency of drinking—3rd trimester						
Every day or almost every day	4	7.7	0	0.0		
3–4 times per week	6	11.5	5	12.8		
1–2 times per week	9	17.3	13	33.3		
1–3 times per month	3	5.8	1	2.6		
1–2 times per 3 months	2	3.8	0	0.0		
Unknown frequency	3	5.8	2	5.1		
Did not drink	25	48.1	18	46.2	.281	
Breastfed (% Yes)^c^	50/52	96.2	35/39	89.7	.223	
Duration of breastfeeding (months)^d^	50	18.2 (16.0)	35	24.5 (18.0)	.097	
Drank alcohol in breastfeeding period (% Yes)^d^	33/50	66.0	22/34	64.7	.903	
Distal maternal risk factors						
Gravidity at intake	55	3.1 (1.8)	41	2.5 (1.3)	.119	
Parity at intake	55	3.0 (1.7)	41	2.3 (1.3)	.028	
Estimated infant gestational age at delivery (weeks)	49	37.4 (2.8)	35	39.2 (1.8)	.002	
Height at interview (cm)	45	155.3 (6.6)	38	155.9 (6.7)	.692	
Weight at interview (kg)	45	55.5 (14.2)	38	59.2 (19.4)	.316	
Occipitofrontal circumference at interview (cm)	45	54.3 (2.2)	38	55.3 (2.7)	.078	
Left upper arm circumference at interview (cm)	45	25.1 (4.3)	37	26.2 (4.8)	.311	
BMI at interview	45	23.0 (5.1)	38	24.2 (6.9)	.367	
Age at interview (yrs)	48	32.9 (8.0)	38	30.1 (7.2)	.086	

^a^Specific alcohol consumption information by trimester was known for *n* = 50 for women without MCM and *n* = 38 for women with MCM.

^b^Among drinkers only.

^c^Breastfeeding information was known for *n* = 52 for women without MCM and *n* = 39 for women with MCM.

^d^Among those who breastfed.

### Comparison of MCM and Non-MCM women with and without nutritional supplementation

Tables A1–A5 in the Appendix summarize a comparison of the 22 MCM participants who received nutritional supplementation and the 19 MCM participants who did not. Women who qualified for supplementation were significantly (*p* < .02) lighter (68.0 *vs.* 50.4 kg), had a lower BMI (27.2 *vs.* 21.1 kg/m^2^), had a smaller upper arm circumference (28.3 *vs.* 23.9 cm) and head circumference (56.6 *vs.* 54.0 cm), and were younger (32.7 *vs.* 27.4 years) (Table A1). There was no significant difference among groups in any of the child physical outcomes at 60 months (Table A2) and one significant difference: motor development at six weeks and 42 months was lower for offspring of the supplemented women (Table A3). Considering KABC results, the offspring of supplemented women performed significantly worse on the KABC sequential short-term memory and number recall (Table A4). Finally (Table A5), there was no significant difference in final diagnosis: the proportion of FAS, PFAS, ARND, ARBD, and Not FASD were similar for both groups. Nutritional supplementation, if it had an effect, may have been to equalize the outcomes by assisting the lighter women with adequate nutrition for similar development as the offspring of the heavier, lower risk, women.

### Final diagnoses at five years of age: MCM vs. non-MCM

A significantly lower proportion of the children of MCM participants was diagnosed with FAS than the non-MCM group (24.4 *vs.* 49.1%, *p* = .01) (Table 2). More of the children with PFAS (19.5 *vs.* 14.5%) and ARND (19.5 *vs.* 14.5%) were born to MCM-participating women than the non-MCM group. One child with ARBD was born to a woman in MCM. Finally, a non-significant, higher proportion of the children without an FASD diagnosis were born to MCM-participating women (34.1 *vs.* 21.8%). The chi-square value of the FASD diagnoses distribution was not statistically significant [*χ*^2^(4) = 7.073, *p* = .132].

**Table 2.** Final diagnoses within the FASD continuum for children at 5 years of age by maternal participation in multifaceted case management.

	Alcohol-exposed without MCM (*n* = 55) *N* (%)	Alcohol-exposed with MCM (*n* = 41) *N* (%)	*p*-Value from *z*-test of proportions
FAS	27 (49.1)	10 (24.4)	.0102
PFAS	8 (14.5)	8 (19.5)	.5261
ARND	8 (14.5)	8 (19.5)	.5261
ARBD	0 (0.0)	1 (2.4)	.3213
Not FASD	12 (21.8)	14 (34.1)	.1892

*χ*^2^(4) = 7.073, *p* = .132.

### Physical characteristics of index children at five years of age by participation in MCM

In Table 3, nine FAS-linked physical trait variables of the index offspring were significantly different between the maternal MCM groups at *p* < .05. All represent more positive outcomes for the MCM offspring group. Head (occipitofrontal) circumference (OFC) centile was larger for the MCM group (21.3 *vs.* 11.9 cm) and fewer of the MCM offspring had OFC measurements that were ≤3rd and ≤10th centile. Four midface measurements were larger for the MCM offspring: inner canthal distance (ICD), interpupillary distance (IPD), palpebral fissure length (PFL), and maxillary arc measurements. There was no significant difference among groups with two of the cardinal facial features of FAS: smooth philtrum and narrow vermilion of the upper lip, both of which are associated with first trimester drinking. Finally, at *p* < .05 significantly fewer minor anomalies were identified (7.7 *vs.* 6.2) in the MCM offspring, and lower mean total dysmorphology scores (12.9 *vs.* 10.5) were characteristic of the offspring of women with MCM. If Bonferroni-adjustment values of significance were applied to Table 3, only three variables were significant: OFC ≤10th centile, OFC ≤3rd centile, and PFL centile.

**Table 3.** Physical characteristics of children at five years of age (60 months) among alcohol exposed pregnancies by mother’s participation in multifaceted case management.

At 60 months	Alcohol-exposed without MCM (*n* = 53)	Alcohol-exposed with MCM (*n* = 38)	*p*
	Mean (*SD*)	Mean (*SD*)	
Height centile	20.2 (25.0)	21.8 (23.5)	.757
Weight centile	17.3 (26.9)	17.4 (21.3)	.989
OFC centile^a^	11.9 (20.6)	21.3 (21.7)	.039
OFC ≤ 3rd centile	62.3	23.7	<.001^b^
OFC ≤ 10th centile	73.6	39.5	.001^b^
Inner canthal distance (ICD) centile	55.5 (18.8)	65.8 (16.7)	.008
Inner pupillary distance (IPD) centile	43.8 (22.7)	58.1 (22.7)	.004
Palpebral fissure length (PFL) centile	11.8 (11.6)	22.2 (17.9)	.001^b^
Smooth philtrum (% Yes)^a^	41.5	44.7	.759
Narrow vermilion (% Yes)^a^	54.7	42.1	.235
Hypoplastic midface (% Yes)	66.0	55.3	.297
Maxillary arc (in cm)	22.5 (1.1)	23.0 (.8)	.024
Mandibular arc (in cm)	23.7 (1.3)	24.1 (1.0)	.150
Strabismus (% Yes)	9.4	7.9	.798
Epicanthal folds (% Yes)	56.6	63.2	.530
Prognathism (% Yes)	0.0	0.0	–
Camptodactyly (% Yes)	9.4	0.0	.051
Number of minor anomalies	7.7 (3.2)	6.2 (2.7)	.018
Total dysmorphology score	12.9 (5.3)	10.5 (5.4)	.035

OFC: occipitofrontal circumference.

^a^Score of 4 or 5 on the South African lip/philtrum guide [67].

^b^Variables are significant at the Bonferroni-adjusted values for this table at *p* < .0026.

Table 4 presents physical trait data for specific FASD diagnoses (FAS, PFAS, and ARND) for children of maternal groups at five years of age. Of the 41 women completing MCM, 27 (65.9%) of their offspring received specific FASD diagnoses, and 14 (34.1%) did not. Children of MCM participants with FAS had a significantly higher average OFC, and fewer had true microcephaly (≤3rd centile) than non-MCM children. MCM participant offspring with PFAS were significantly more likely to have a smooth philtrum. Approaching significance (.05 ≤ *p* ≤ .10) were larger heads, larger ICD, and larger PFL. Finally, for the ARND group, OFC, ICD, and IPD were all significantly larger for the MCM participant offspring. Indicators of better brain development were present in the MCM offspring within each specific diagnosis category.

**Table 4.** Child physical traits by specific FASD diagnosis at 5 years of age (60 months) by maternal participation in multifaceted case management.

(A) Children with FAS			
At 60 months	Alcohol-exposed without MCM^a^ (*n* = 26)	Alcohol-exposed with MCM^a^ (*n* = 9)	*p*
	Mean (*SD*)	Mean (*SD*)	
OFC centile	1.4 (1.0)	2.7 (2.5)	.036
OFC ≤ 3rd centile	96.2	66.7	.017
Inner canthal distance (ICD) centile	56.6 (17.4)	61.6 (19.9)	.483
Inner pupillary distance (IPD) centile	37.3 (19.7)	36.3 (12.4)	.887
Palpebral fissure length (PFL) centile	5.9 (5.9)	5.9 (7.9)	.999
Smooth philtrum (% Yes)	73.1	77.8	.781
Narrow vermilion (% Yes)	69.2	77.8	.625
Number of minor anomalies	9.6 (2.7)	9.2 (1.6)	.713
Total dysmorphology score	16.8 (3.4)	16.8 (3.2)	.986
(B) Children with PFAS			
	Alcohol-exposed without MCM (*n* = 7)	Alcohol-exposed with MCM (*n* = 8)	
At 60 months	Mean (*SD*)	Mean (*SD*)	*p*
OFC centile	12.4 (10.9)	36.3 (31.3)	.079
Inner canthal distance (ICD) centile	55.9 (12.4)	68.0 (10.0)	.056
Inner pupillary distance (IPD) centile	39.0 (12.1)	54.1 (24.8)	.167
Palpebral fissure length (PFL) centile	6.0 (6.8)	19.6 (17.0)	.070
Smooth philtrum (% Yes)	28.6	87.5	.020
Narrow vermilion (% Yes)	71.4	62.5	.714
Number of minor anomalies	7.6 (1.1)	7.3 (.7)	.515
Total dysmorphology score	12.9 (1.8)	12.1 (2.4)	.514
(C) Children with ARND			
	Alcohol-exposed without MCM (*n* = 8)	Alcohol-exposed with MCM (*n* = 8)	
At 60 months	Mean (*SD*)	Mean (*SD*)	*p*
OFC centile	4.5 (4.3)	19.3 (13.3)	.010
OFC ≤ 3rd centile	50.0	25.0	.302
OFC ≤ 10th centile	87.5	37.5	.039
Inner canthal distance (ICD) centile	46.3 (22.8)	70.8 (12.7)	.019
Inner pupillary distance (IPD) centile	44.1 (23.5)	72.4 (15.8)	.014
Palpebral fissure length (PFL) centile	17.0 (12.5)	21.8 (13.8)	.482
Smooth philtrum (% Yes)	0.0	25.0	.131
Narrow vermilion (% Yes)	37.5	0.0	.055
Number of minor anomalies	6.8 (2.6)	4.9 (2.8)	.187
Total dysmorphology score	10.5 (3.1)	8.4 (5.3)	.341

^a^All mothers in both categories had AUDIT scores > 8 and were considered hazardous drinkers.

### Neurobehavioral traits of children compared by participation in MCM and age

Neurobehavioral traits on the BSID-III are presented in Table 5 for children at 6 weeks to 42 months. Offspring of the MCM group scored lower on all domains (cognitive, language, motor, and social/emotional) up to 42 months. The MCM offspring were significantly lower in specific categories of functioning on most all domains (cognitive, language, motor, and social emotional) at 18 months and younger, but not at 42 months.

**Table 5.** BSID-III percentile rank of children by mother’s multifaceted case management participation.

	Alcohol-exposed without MCM	Alcohol-exposed with MCM	*p*
	Mean (*SD*)	Mean (*SD*)	
Cognitive percentile rank at 6 weeks^a^	57.0 (31.4)	20.2 (15.4)	<.001
Cognitive percentile rank at 9 months^b^	49.6 (30.2)	47.9 (26.5)	.811
Cognitive percentile rank at 18 months^c^	42.0 (26.2)	18.4 (14.1)	<.001
Cognitive percentile rank at 42 months^d^	31.3 (14.2)	24.0 (14.6)	.301
Language percentile rank at 6 weeks^a^	36.2 (28.1)	14.4 (12.4)	<.001
Language percentile rank at 9 months^b^	32.2 (28.3)	15.6 (22.1)	.012
Language percentile rank at 18 months^c^	28.0 (17.9)	19.8 (14.4)	.057
Language percentile rank at 42 months^d^	31.5 (15.2)	17.8 (12.5)	.056
Motor percentile rank at 6 weeks^a^	69.2 (26.0)	38.2 (20.9)	<.001
Motor percentile rank at 9 months^b^	39.8 (30.6)	25.9 (17.1)	.037
Motor percentile rank at 18 months^c^	39.6 (29.8)	28.9 (19.9)	.114
Motor percentile rank at 42 months^d^	68.6 (21.9)	46.0 (24.7)	.057
Social/emotional percentile rank at 6 weeks^a^	46.7 (26.7)	42.4 (24.2)	.527
Social/emotional percentile rank at 9 months^b^	52.2 (32.6)	35.8 (30.1)	.039
Social/emotional percentile rank at 18 months^c^	54.7 (31.2)	30.4 (22.6)	.001
Social/emotional percentile rank at 42 months^d^	50.2 (20.3)	33.8 (19.2)	.093

^a^*N* = 43 for alcohol-exposed with MCM; *N* = 22 for alcohol-exposed with MCM.

^b^*N* = 43 for alcohol-exposed with MCM; *N* = 27 for alcohol-exposed with MCM.

^c^*N* = 42 for alcohol-exposed with MCM; *N* = 25 for alcohol-exposed with MCM.

^d^*N* = 10 for alcohol-exposed with MCM; *N* = 8 for alcohol-exposed with MCM.

The KABC results (Table 6) indicated that by age 5, the offspring groups were virtually identical in their performance in all domains: global, short- and long-term memory, and visual processing. No average percentile ranks were above 37, indicating poor performance for both groups. Only long-term storage of information and retrieval percentile rank approached significance, and this *p*-value may be an artifact of multiple comparisons.

**Table 6.** KABC-II age-adjusted percentile rank among children by mother’s multifaceted case management participation.

	Alcohol-exposed without MCM (*n* = 42)	Alcohol-exposed with MCM^a^ (*n* = 32)	*p*
	Mean (*SD*)	Mean (*SD*)	
Global percentile rank	10.9 (16.2)	9.2 (12.5)	.574
Sequential short term memory percentile rank	23.6 (20.8)	20.6 (20.2)	.474
Number recall percentile rank	36.3 (28.6)	35.5 (29.1)	.263
Word order recall percentile rank	18.6 (13.8)	14.8 (14.8)	.993
Simultaneous visual processing percentile rank	8.0 (15.6)	5.0 (7.9)	.865
Triangles percentile rank	12.2 (16.4)	13.5 (16.7)	.208
Pattern reasoning percentile rank	20.3 (22.2)	12.0 (15.6)	.795
Conceptual thinking percentile rank	8.8 (11.6)	9.9 (18.2)	.880
Long term storage and retrieval percentile rank	25.0 (21.6)	25.1 (19.5)	.077
Atlantis percentile rank	36.6 (27.6)	33.4 (24.8)	.494
Rebus percentile rank	18.9 (18.8)	22.9 (23.3)	.428

^a^Due to end of funding for the grant at the intervention site, nine of the offspring of women participating in MCM were not tested and the neurobehavioral component of their diagnosis were based on their Bayley scores.

### Logistic regression analysis

Regression analysis compares the association of MCM to selected child developmental outcomes (Table 7). Children born to alcohol-using women without MCM (standard care), were 3.15 times more likely to have FAS (*p* = .034, 95% CI = 1.09–9.08) than MCM offspring. OFC in the offspring of MCM participants was also associated with a positive outcome. The odds of a head circumference ≤3rd centile (*p* < .001, 95% CI = 2.09–13.49), and of an OFC ≤10th centile (*p* = .001, 95% CI = 1.75–10.42) were 5.32 and 4.27 greater for the non-MCM offspring. Finally, not receiving MCM was associated with a decrease in the length of eye openings (palpebral fissure length—PFL) over that of children of women in MCM (OR = 2.46, *p* = .039, 95% CI = 1.05–5.78). Smaller head circumference and PFL are considered direct measures of poor brain growth and development.

**Table 7.** Binary logistic regression associating FAS diagnosis and select physical features as a function of multifaceted case management participation status.

	*B*	SE	Sig	Odds ratio	95% CI	
					Lower	Upper
Predicting FAS (*n* = 63)						
Not in MCM *vs.* in MCM (reference)	1.147	.540	.034	3.15	1.093	9.081
Constant	−.336	.414	.416	.714	–	–
Predicting OFC ≤3rd centile (*n* = 91)						
Not in MCM *vs.* in MCM (reference)	1.671	.475	<.001	5.317	2.094	13.496
Constant	−1.170	.382	.002	.310	–	–
Predicting OFC ≤10th centile (*n* = 91)						
Not in MCM *vs.* in MCM (reference)	1.452	.455	.001	4.271	1.750	10.424
Constant	−.427	.332	.198	.652	–	–
Predicting PFL ≤3rd centile (*n* = 91)						
Not in MCM *vs.* in MCM (reference)	.900	.436	.039	2.461	1.047	5.782
Constant	−.318	.329	.332	.727	–	–

Another comparison in Table 8 assessed the difference in the offspring of the two groups who reported 3rd trimester drinking. In three of the comparisons, FAS diagnosis, OFC ≤ 3rd, and OFC ≤10th, the offspring of non-participants were (respectively OR = 6.0, 4.9, and 6.5) more likely to have these less desirable traits.

**Table 8.** Binary logistic regression associating FAS diagnosis and select physical features by multifaceted case management participation status among those who drank in 3rd trimester.

	*B*	SE	Sig	Odds ratio	95% CI	
					Lower	Upper
Predicting FAS (*n* = 29)						
Not in MCM *vs.* in MCM (reference)	1.792	.876	.041	6.00	1.079	33.378
Constant	−.182	.606	.763	.833	–	–
Predicting OFC ≤3rd centile (*n* = 42)						
Not in MCM *vs.* in MCM (reference)	1.600	.670	.017	4.952	1.332	18.414
Constant	−.773	.494	.117	.462	–	–
Predicting OFC ≤10th centile (*n* = 42)						
Not in MCM *vs.* in MCM (reference)	1.877	.720	.009	6.531	1.592	26.788
Constant	−.318	.465	.493	.727	–	–
Predicting PFL ≤3rd centile (*n* = 42)						
Not in MCM *vs.* in MCM (reference)	.116	.836	.890	1.123	.218	5.777
Constant	−1.674	.629	.008	.188	–	–

## Discussion

Binge drinking is normative and common on weekends among women of childbearing age in the two communities engaged in this study [20,28]. The population of the two study communities was ∼50,000 predominantly mixed-race, ‘Coloured’ individuals in each. The social, cultural, and economic conditions are similar in the two sub-regions, and drinking frequently continues through much of the first trimester for a majority of the women who are drinkers. Many women drink throughout the second trimester, and some throughout the entire pregnancy [4,6]. There are few outlets for recreation for the workers on the farms, orchards, vineyards and small industries, and group drinking on Friday and Saturday night is a major and commonly accepted mode of relaxation and recreation. The result is many alcohol-exposed pregnancies and the highest known rates of FASD recorded in the world to date, rates of 28–31% among active case ascertainment, first grade students in population-bases studies [4,6,27,73].

When the first studies were begun on the epidemiology of FAS in one of these two communities in 1997, it became obvious that the impact of the socio-economic conditions on the families, women, and mothers of the population had to be addressed to reduce the rate of binge drinking and the resultant high rates of FASD. The first comprehensive studies of maternal risk for FASD in these communities indicated the need for a multifaceted approach to address the educational and support needs of the women at the highest risk of having a child with FASD. The MCM approach was the impetus for developing and implementing the prevention of FASD and the evaluation in this manuscript. Furthermore, to our knowledge, this is the first attempt ever to evaluate an approach to FASD prevention by assessing outcome/efficacy *via* the specific FASD diagnoses and severity of a broad array of both physical and neurobehavioral child developmental traits linked to prenatal alcohol exposure and FASD.

All the women in this evaluation of MCM were recruited from two adjacent regions of the WCP which were of virtually identical socio-economic status and shared a common Western Cape culture. This similarity made group comparison based on MCM participation substantively quite meaningful. The comparisons in this analysis indicated that all the women in these evaluations were hazardous drinkers as indicated by their AUDIT and T-ACE scores at recruitment. Not only were women who participated in MCM identified to be at higher risk on the AUDIT for having a child with FASD, some additional proximal variables of alcohol consumption, such as prevalence and frequency of drinking in the first trimester and throughout pregnancy were significantly higher than the comparison group or there was no significant difference between MCM and non-MCM groups. Furthermore, a comparison of the groups on distal measures of risk for FASD indicated that only one trait, parity, was significant. Therefore, in comparison with other high risk, non-MCM participants, MCM was associated with infants who were physically larger, physically better developed, and had less FASD-specific dysmorphology when compared to alcohol-exposed infants born to women not enrolled in MCM. One question that we cannot answer is if the women who agreed to participate in MCM were self-motivated and ready to change [33,74,75]. If so, were they better candidates for this MCM evaluation than if selected randomly? Since we did not administer a readiness for change evaluation at recruitment or intake, the answer is elusive.

### Nutritional enhancement for women with low body mass

One unique aspect of the MCM program was a nutritional enhancement provision for those women who were underweight. Providing daily, multi-vitamin supplements to low BMI women participating in MCM for prenatal consumption may have provided an advantage to MCM women [76] even though the mean weight and BMI were not significantly different at recruitment between the groups. In this population, where a high percentage of women are undernourished in the childbearing ages [77,78], low maternal weight and BMI were associated previously with a higher risk for FASD [5–7,20,62,63,79]. A comparison of child outcomes from supplemented *vs.* non-supplemented women was summarized in the Appendix (Tables A1–A5). Supplementation did not result in larger children or better neurobehavior relative to the non-supplemented women. One might speculate that without the maternal supplementation, the children of the lower BMI women would have been significantly smaller, but further research is needed on more women who enter prenatal care earlier and receive nutritional supplementation for most of the prenatal period. Nevertheless, findings of no significant difference between the supplemented women and the other MCM women strengthen the case for a fair evaluation of the efficacy of MCM.

### Head circumference and neurobehavioral traits of the children in MCM and not in MCM

Although the cognitive and behavioral performance was similar for the groups of children, offspring of women in MCM had significantly larger head circumferences than other alcohol-exposed children without MCM. Head circumference at birth and the early years of life is a measurement that often indicates better prenatal and childhood brain growth and development and potential neurobehavioral development later in life [80,81]. A greater proportion of alcohol-exposed children without MCM had true microcephaly, OFC ≤3rd centile, which is two or more standard deviations below the mean, than did children with MCM. Therefore, the trajectory of cognitive performance of the children without MCM may suffer a greater relative decline than the offspring of MCM participants as the children age [82,83]. Overall, the analyses indicated that a reduced likelihood of a severe diagnosis within the continuum of FASD, especially of FAS, was associated with MCM. The verdict is out on neurobehavioral development, for significant improvement in cognition and behavior was not demonstrated, and all children evaluated had below average performance. Neurobehavioral outcomes should, however, be followed over time, for cognitive performance varies greatly as children age, and recent studies report that better physical indicators at an earlier age bode well for improved cognitive development and function later [80,81]. Before 5 years of age, assessments for children are primarily measures of developmental milestones and not measures of advanced cognitive abilities [84,85], and the children of women participating in MCM performed worse than the offspring of the non-MCM group. Testing at age 8 and above with more sophisticated, age-appropriate tests will be better indicators of the abilities of children from the two groups.

### Alcohol consumption and multiple child outcomes from this MCM evaluation

Participation in MCM was associated with a significant reduction in prenatal alcohol consumption in previous evaluations. Mean drinks per weekend, drinking days per week, and AUDIT scores were all reduced significantly six months into MCM [58,61]. Therefore, a significant reduction in antenatal drinking and often during the first year of life *via* breastmilk when brain growth was vulnerable [86], is likely associated with the major differences in group means, and in the lower odds ratios for adverse outcomes (small head circumference, midface hypoplasia, and a diagnosis of FAS in the MCM offspring). The diagnosis of FAS is, by definition, characterized by the most depressed physical growth and development, more dysmorphology—especially in the brain and midface [66,87,88]. Children with FAS are generally exposed to the most alcohol over the duration of the pregnancy, and OFC and brain development can be affected by alcohol in all trimesters. Two previous publications on this MCM process reported an association between MCM and a reduction in drinking and abstinence [57,58]. Tables 7 and 8 confirmed this association by comparing the women of both groups, and even those who continued to drink in the third trimester. The significant odds ratio indicated 6–6.5 times of increased risk for FAS and microcephaly in children whose mothers did not receive MCM. Overall, a non-statistically significant (*p* = .189) higher proportion of the offspring from MCM women wasdiagnosed as Not-FASD (34 *vs.* 22%). Therefore, one possible conclusion is that the lower severity of diagnosis in children from MCM was commonly associated with reductions in alcohol exposure in the later stages of pregnancy. Women in MCM drank less per drinking day, especially in the second and third trimesters, and this was associated with significantly improved OFC and PFL, and significantly fewer were diagnosed as FAS.

Other positive outcomes might have resulted from MCM. General health and social advice and encouragement for better maternal health practices were communicated empathically *via* MCM and may have contributed to the measured outcomes. In the previous evaluation, women were reported as significantly happier in MCM over time, and this may have helped improve child outcomes [57,58]. Certainly, the general improvement in knowledge of fetal and child development, emotional support, advice on important life choices, and more access to, and the support of, health care professionals provided by MCM project staff was consistent with themes in the prenatal screening and intervention literature [38,43,44]. Many women with hazardous alcohol use in the WCP commonly suffer from multiple problems affecting quality of life: stress, anxiety and depression during pregnancy, and sexual or domestic violence [89]. MCM was well accepted and appreciated by most of the participants from an otherwise underserved population.

### FASD policy for prevention in South Africa

The continued high prevalence of FASD in South Africa indicates that there is a need for coordinated prevention efforts on all three levels of prevention and early diagnosis of affected individuals as well as interventions to manage the needs of individuals with FASD [90–93]. Such a comprehensive approach that also takes social determinants of disease into account, calls for specific FASD policies. Despite the extent of the problem with FASD in South Africa, there is no national policy to ensure effective and sustainable services or to create a framework to implement a system for integrated care for individuals with FASD [90,94]. Currently, different government departments address FASD in a generic manner according to their departmental policies and guidelines. This leads to uncoordinated and ineffective services being delivered ‘in silos’ [95].

With the per capita alcohol consumption in South Africa the highest in Africa, it is recommended that FASD policy should also address alcohol policy, specifically the availability of alcohol, maternal alcohol use in pregnancy, and the roles and responsibilities of stakeholders. Other important aspects for FASD policy are that the guiding principles should be holistic, multi-sectoral, human rights based, and culturally sensitive. An FASD policy should also be education-, health-, and community/socially-related [90,92].

### Strengths and limitations of this study

This study has several strengths. First, this is the first study, to our knowledge, that has assessed, in detail, the effectiveness of the prenatal intervention to prevent FASD by employing dysmorphology examinations, neurobehavioral testing, and a formal diagnostic evaluation of FASD in the offspring of women in MCM. Direct child outcomes were assessed to evaluate the efficacy of MCM and enhance normal development in children with PAE. Second, the children’s growth and development were followed over time for both groups of at risk, heavy drinking women. There are few FASD prevention studies with controls that assessed child developmental indicators over time [50,81–83,96]. Third, the women in the two groups were well matched on both proximal risk factors (alcohol use in a population that has little co-morbid use of other drugs) and distal risk factors at intake. Fourth, we have done our ethical best and provided a novel intervention, nested and blinded, within a comparative study of the development of children with FASD and controls, to assist as many of those who were the most hazardous drinkers as we could. Obviously, the lack of random assignment causes problems with interpretation of causality, but it was a proper and appropriate intervention for an applied, on the ground public health study. It was a realistic, real world attempt to answer two major priorities for FASD intervention research raised by Hankin et al. [97]: generate a program that reduces barriers to care and determine which programs might be most successful.

There are also limitations to this study. First, the sample size was relatively small and was somewhat limited in statistical power. Second, due to some missed appointments and funding limitations, there were missing data at each time point, and for three dropouts of MCM (6.8%), there were insufficient data for inclusion in this analysis. Third, by design, the study was not randomized. We provided MCM only to the highest risk, heaviest drinking women in one region of our research activities, which made evaluation more challenging. Within our resource limitations, we provided services to as many high-risk women in accordance with our public health research paradigm, to evaluate the impact of MCM. Fourth, while the final diagnosis of the children was performed with the highly-rated, revised IOM diagnostic criteria and processes [73], there was some variance in the age at which all testing and dysmorphology exams were provided. However, since all measures used were applied after appropriate benchmarks had been reached and age-adjusted scores/centiles were utilized, these age/timing problems have been corrected in the analysis. Fifth, the fact that the non-MCM participants were not from the exact same geographic location could be considered a limitation. But given the striking similarities in social, economic, and cultural conditions in these two sub-populations of the WCP, the danger of spurious results is minimized. Finally, a sixth limitation is that no biological markers for alcohol or other drug use were employed in this study. Consequently, these self-reported data may not adequately reflect the actual magnitude of alcohol and other drug use in either group. But, reporting in this population has proven to be accurate in the binary sense in other studies/samples [98].

## Conclusion

Multi-faceted Case Management, using elements of Motivational Interviewing, Community Reinforcement Approach, and empathic social work skills can provide significant improvement in child outcomes that are measurable at five years of age and likely in later years.

## APPENDIX

**Table A1.** Maternal risk factors among alcohol-exposed pregnancies by maternal multifaceted case management participation and by nutritional supplementation.

	Alcohol-exposed with MCM not supplemented (*n* = 19)	Alcohol-exposed with MCM supplemented (*n* = 22)	*p*
	Mean (*SD*)	Mean (*SD*)	
Alcohol			
T-ACE at intake	4.2 (.9)	3.8 (1.2)	.312
AUDIT score at intake	20.2 (6.9)	19.3 (8.1)	.709
Estimated Month Enrolled in MCM	4.9 (2.2)	4.7 (1.6)	.711
Drank in 1st trimester (% Yes)^a^	94.7	94.7	–
Usual number of drinks per drinking day—1st trimester	5.4 (3.0)	6.3 (4.6)	.502
Usual number of drinks per drinking day—1st trimester^b^	5.7 (2.8)	6.6 (4.5)	.472
Usual frequency of drinking—1st trimester			
Every day or almost every day	0.0	0.0	
3–4 times per week	47.4	50.0	
1–2 times per week	47.4	40.0	
1–3 times per month	0.0	0.0	
1–2 times per 3 months	0.0	0.0	
Unknown frequency	0.0	5.0	.780
Did not drink	5.3	5.0	
Drank in 2nd trimester (% Yes)^a^	89.5	84.2	.631
Usual number of drinks per drinking day—2nd trimester	5.2 (3.6)	5.6 (5.3)	.812
Usual number of drinks per drinking day—2nd trimester^b^	5.8 (3.2)	6.6 (5.2)	.603
Usual frequency of drinking—2nd trimester			
Every day or almost every day	0.0	0.0	
3–4 times per week	36.8	35.0	
1–2 times per week	47.4	40.0	
1–3 times per month	5.3	5.0	
1–2 times per 3 months	0.0	0.0	
Unknown frequency	10.5	5.0	.872
Did not drink	0.0	15.0	
Drank in 3rd trimester (% Yes)^a^	52.6	52.6	–
Usual number of drinks per drinking day—3rd trimester	2.5 (3.3)	2.6 (4.9)	.969
Usual number of drinks per drinking day—3rd trimester^b^	4.8 (3.0)	5.2 (6.0)	.868
Usual frequency of drinking—3rd trimester			
Every day or almost every day	0.0	0.0	
3–4 times per week	15.8	10.0	
1–2 times per week	31.6	35.0	
1–3 times per month	5.3	0.0	
1–2 times per 3 months	0.0	0.0	
Unknown frequency	47.4	45.0	
Did not drink	0.0	10.0	.516
Breastfed (% Yes)^c^	89.5	90.0	.957
Duration of breastfeeding (months)^d^	26.4 (18.9)	27.2 (17.2)	.374
Drank alcohol in breastfeeding period (% Yes)^d^	58.8	70.6	.473
Distal maternal risk factors			
Gravidity at intake	2.9 (1.5)	2.2 (1.1)	.064
Parity at intake	2.6 (1.6)	2.0 (.9)	.093
Height at interview (cm)	157.1 (7.6)	154.7 (5.5)	.274
Weight at interview (kg)	68.0 (22.0)	50.4 (11.3)	.004
Occipitofrontal circumference at interview (cm)	56.6 (2.9)	54.0 (1.7)	.002
Upper left arm circumference at interview (cm)	28.3 (4.8)	23.9 (3.7)	.004
BMI at interview	27.2 (7.5)	21.1 (4.6)	.004
Age at interview (yrs)	32.7 (8.1)	27.4 (5.1)	.019

^a^Specific alcohol consumption information by trimester was known for *n* = 19 for women without supplementation and *n* = 19 for women with supplementation.

^b^Among drinkers only.

^c^Breastfeeding information was known for *n* = 19 for women without supplementation and *n* = 20 for women with supplementation.

^d^Among those who breastfed.

**Table A2.** Physical characteristics of children at 60 months of age among alcohol exposed pregnancies by mother’s multifaceted case management participation and by nutritional supplementation.

At 60 months	Alcohol-exposed with MCM not supplemented (*n* = 19)	Alcohol-exposed with MCM supplemented (*n* = 19)	*p*
	Mean (*SD*)	Mean (*SD*)	
Height centile	19.4 (25.9)	24.2 (21.3)	.538
Weight centile	15.4 (23.9)	19.4 (18.7)	.574
OFC centile	18.8 (17.8)	23.7 (25.3)	.491
OFC ≤ 3rd centile	21.1	26.3	.703
OFC ≤ 10th centile	47.4	31.6	.319
Inner canthal distance (ICD) centile	62.8 (20.8)	68.8 (11.0)	.278
Inner pupillary distance (IPD) centile	58.8 (23.2)	57.4 (22.8)	.845
Palpebral fissure length (PFL) centile	22.6 (18.6)	21.8 (17.6)	.887
Smooth philtrum (% Yes)	52.6	36.8	.328
Narrow vermilion (% Yes)	31.6	52.6	.189
Hypoplastic midface (% Yes)	63.2	47.4	.328
Maxillary arc (in cm)	23.2 (.8)	22.8 (.8)	.155
Mandibular arc (in cm)	24.4 (1.0)	23.8 (.9)	.055
Strabismus (% Yes)	10.5	5.3	.547
Epicanthal folds (% Yes)	57.9	68.4	.501
Prognathism (% Yes)	0.0	0.0	–
Camptodactyly (% Yes)	0.0	0.0	–
Number of minor anomalies	6.4 (2.8)	5.9 (2.7)	.598
Total dysmorphology score	11.0 (5.9)	9.9 (4.9)	.552

**Table A3.** BSID-III percentile rank of children by mother’s multifaceted case management participation and by nutritional supplementation.

	Alcohol-exposed with MCM not supplemented (*n* = 15)	Alcohol-exposed with MCM supplemented (*n* = 17)	*p*
	Mean (*SD*)	Mean (*SD*)	
Cognitive percentile rank at 6 weeks^a^	19.9 (14.4)	22.1 (16.7)	.718
Cognitive percentile rank at 9 months^b^	44.9 (27.0)	50.8 (23.9)	.461
Cognitive percentile rank at 18 months^c^	24.2 (15.7)	20.2 (17.0)	.474
Cognitive percentile rank at 42 months^d^	25.8 (13.1)	32.4 (14.7)	.413
Language percentile rank at 6 weeks^a^	10.2 (10.5)	18.5 (16.0)	.132
Language percentile rank at 9 months^b^	20.3 (28.9)	23.1 (26.2)	.748
Language percentile rank at 18 months^c^	22.9 (19.5)	22.5 (13.6)	.943
Language percentile rank at 42 months^d^	22.2 (13.3)	27.9 (17.9)	.542
Motor percentile rank at 6 weeks^a^	46.0 (20.0)	32.1 (16.4)	.046
Motor percentile rank at 9 months^b^	36.7 (25.7)	29.8 (21.3)	.363
Motor percentile rank at 18 months^c^	26.3 (22.7)	32.1 (16.3)	.391
Motor percentile rank at 42 months^d^	35.0 (28.8)	62.6 (19.7)	.046
Social/emotional percentile rank at 6 weeks^a^	38.3 (18.0)	35.5 (27.5)	.754
Social/emotional percentile rank at 9 months^b^	38.5 (31.7)	39.9 (32.6)	.892
Social/emotional percentile rank at 18 months^c^	35.2 (27.0)	34.8 (25.7)	.970
Social/emotional percentile rank at 42 months^d^	36.6 (24.4)	43.4 (24.0)	.611

^a^*N* = 11 for not supplemented; *N* = 22 for supplemented.

^b^*N* = 19 for not supplemented; *N* = 22 for supplemented.

^c^*N* = 16 for not supplemented; *N* = 19 for supplemented.

^d^*N* = 5 for not supplemented; *N* = 10 for supplemented.

**Table A4.** KABC-II age-adjusted percentile rank among children by mother’s multifaceted case management participation and by nutritional supplementation.

	Alcohol-exposed with MCM not supplemented (*n* = 15)	Alcohol-exposed with MCM supplemented (*n* = 17)	*p*
	Mean (*SD*)	Mean (*SD*)	
Global percentile rank	9.6 (11.2)	8.8 (14.0)	.776
Sequential short term memory percentile rank	27.9 (21.3)	13.8 (16.9)	.036
Number recall percentile rank	52.0 (27.7)	20.0 (21.3)	<.001
Word order recall percentile rank	14.8 (14.6)	14.9 (15.6)	.890
Simultaneous visual processing percentile rank	5.0 (8.6)	5.0 (7.3)	.370
Triangles percentile rank	12.3 (14.0)	14.6 (19.3)	.887
Pattern reasoning percentile rank	9.8 (12.9)	14.1 (17.9)	.430
Conceptual thinking percentile rank	12.6 (21.2)	7.4 (15.0)	.407
Long term storage and retrieval percentile rank	22.4 (15.9)	27.6 (22.5)	.470
Atlantis percentile rank	29.3 (23.4)	37.1 (26.1)	.533
Rebus percentile rank	22.9 (21.3)	22.8 (25.7)	.989

**Table A5.** Final specific diagnoses within the FASD continuum for children at 5 years of age by maternal multifaceted case management participation and by nutritional supplementation.

	Alcohol-exposed with MCM not supplemented (*n* = 19) *N* (%)	Alcohol-exposed with MCM supplemented (*n* = 22) *N* (%)	*p*-Value from *z*-test of proportions
FAS	6 (31.6)	4 (18.2)	.3322
PFAS	3 (15.8)	5 (22.7)	.5824
ARND	4 (21.1)	4 (18.2)	.8205
ARBD	0 (0.0)	1 (4.5)	.3199
Not FASD	6 (31.6)	8 (36.4)	.7518

*χ*^2^(4) = 1.977, *p* = .740.
